# Perception and utilization of prevention of mother-to-child transmission of human immunodeficiency virus (HIV) services among women living with HIV

**DOI:** 10.18332/ejm/140454

**Published:** 2021-09-16

**Authors:** Anifat O. Saka, Chiemerigo A. Onyeneho, Chizoma M. Ndikom

**Affiliations:** 1 Department of Nursing, Faculty of Clinical Sciences, College of Medicine, University of Ibadan, Ibadan, Nigeria

**Keywords:** HIV, prevention, transmission, utilization, perception, mother-to-child

## Abstract

**INTRODUCTION:**

Mother-to-child transmission is the major route of pediatric Human Immunodeficiency Virus (HIV) infection accounting for 90% of childhood HIV infection. Poor utilization of prevention of mother-to-child transmission (PMTCT) of HIV services has been shown in this situation. Hence, the study assessed the perception and utilization of PMTCT services among Women Living with HIV (WLHIV).

**METHODS:**

A cross-sectional research design was used with a purposive sampling technique to select 182 WLHIV within reproductive age attending President’s Emergency Plan for Acquired Immunodeficiency Syndrome (AIDS) Relief (PEPFAR)/AIDS Prevention Initiative in Nigeria (APIN) clinic in two secondary Health facilities in Ibadan, Oyo State. A validated structured questionnaire was used for data collection. Descriptive and inferential statistics were used for data analysis.

**RESULTS:**

The mean age of the women was 37.0±6.5 years. Majority (74.2%) of the respondents had good knowledge on PMTCT of HIV, positive perception (89%) towards PMTCT services while only 42.9% of the respondents have utilized PMTCT services during pregnancy. However, some of the challenges to use of PMTCT services identified by the respondents were stigma (16.5%), discrimination (15.4%), financial constraint (11.5%) and non-involvement of partner (8.2%). There was a significant association between level of knowledge and PMTCT services utilization (χ^2^=6.244, p=0.012).

**CONCLUSIONS:**

There is need for improvement of knowledge and perception of HIV, MTCT and PMTCT among women through counseling and antenatal education, thereby increasing PMTCT services uptake. Partner involvement, good quality PMTCT services and lack of discrimination of people living with HIV in our society should be encouraged, hence promoting the utilization of PMTCT services.

## INTRODUCTION

Human Immunodeficiency Virus (HIV) is currently a major global public health crisis^1^. Although, successes have been recorded in reducing the incidence and mortality of HIV/ AIDS across the globe, the rates of new HIV infections remain disproportionately high across Sub-Saharan Africa, particularly among young women^2^. Sub-Saharan Africa accounts for 9 in 10 of women and children living with HIV^3^. The rate of mother-to-child transmission (MTCT) of HIV in Nigeria is unacceptably high, accounting for 30% of the global burden of mother-to-child-transmission of HIV^2^. In 2017, 220000 children (0–14 years) in Nigeria were living with HIV^4^. The high burden of MTCT of HIV in Nigeria has been linked with high prevalence of HIV in women of reproductive age and low prevention of MTCT coverage^5^.

MTCT of HIV is the transmission of the virus from mother living with HIV infection to her child during pregnancy, labor, delivery or breastfeeding^6^. These have been identified as major routes accounting for 90% of childhood HIV infection^6^. PMTCT is a recommended approach for virtual elimination of pediatric HIV to help curb the proportion of MTCT of HIV^7^ and also one of the greatest public health achievements in recent times. The World Health Organization (WHO) promotes a comprehensive approach to PMTCT programs which include preventing new HIV infections among women of childbearing age, preventing unintended pregnancies among women living with HIV (WLHIV), preventing HIV transmission from a woman living with HIV to her baby and providing appropriate treatment, care and support to mothers living with HIV and their children and families^5^. Effective PMTCT programs require women and their infants to receive a cascade of interventions or services such as uptake of antenatal services and HIV testing during pregnancy, use of antiretroviral treatment (ART) by pregnant women living with the virus, safe childbirth practices and appropriate infant feeding, uptake of infant HIV testing and other post-natal healthcare services^8^.

Utilization of PMTCT services is a means of reducing the incidence of MTCT of HIV among WLHIV. Studies have reported a link between low incidence of MTCT or possibility of a child contracting HIV from the mother and utilization of PMTCT services^9,10^. PMTCT services are being provided in healthcare institutions across the globe as well as in Nigeria. Despite the provision of these services, there is still poor utilization of PMTCT services^11^.

According to National Agency for Control of AIDS (NACA, 2017), in the first quarter of 2017 only 59010 out of the estimated 246896 children living with HIV in Nigeria were on treatment; among the pregnant women living with HIV, approximately 50000 received antiretroviral drugs to prevent MTCT of HIV, and in mid-2017, antiretroviral treatment coverage was 25% of children living with HIV. It was also reported that the highest unmet need for PMTCT in Nigeria was in 12 states including Oyo state. The director of NACA (as cited in Daily Trust, 2017) stated that in Oyo state 10000 pregnant women are living with HIV; there are gaps between infected pregnant women and those receiving treatment and about 50 people contract HIV in the state on daily basis. There was also inadequate early infant diagnosis coverage, viral load testing services and weak referral linkage (NACA, 2017)^12^.

Factors that have been associated in the utilization of PMTCT services include knowledge, perception, and accessibility of PMTCT services amongst others. Several studies identified that knowledge and perception about health services are basic factors and determinants of the acceptability of such services^13^. Research has shown that the women’s knowledge on PMTCT services, ART and MTCT varied between good and poor^6,7,9,13,14^. Women with adequate knowledge of MTCT and PMTCT have been reported to be significant in PMTCT services uptake^9,15,16^ while women with inadequate knowledge of MTCT and PMTCT services were more likely to be ART defaulters^9^. Hence, knowledge of pregnant women on MTCT of HIV has implications for child HIV acquisition^6^. Since previous studies have divergent findings and the studies were not carried out in the study are, there was a need to evaluate the situation in the study area.

Furthermore, the perceptions of people about HIV and MTCT will have an implication in their utilization of PMTCT services. This is supported by some studies which reported that perceptions of women have been linked to the utilization of PMTCT services^10^. Hence, there is a need to assess the knowledge, perception and utilization of PMTCT of HIV services among WLHIV.

The study specific objectives were: to assess women’s level of knowledge on prevention of mother to child transmission of HIV; to elicit the women’s perception about prevention of mother to child transmission of HIV (PMTCT) programs and services; to investigate the utilization of PMTCT services among HIV infected women; and to identify the perceived challenges to PMTCT services utilization.

## METHODS

A cross-sectional research design was used with a purposive sampling technique to select a sample size of 182 WLHIV within reproductive age. This study was conducted for three months (July 2018 – October 2018). A validated structured questionnaire (r=0.67) was used to collect data on knowledge, perception, utilization and perceived challenges to PMTCT of HIV services from the women attending PEPFAR/APIN clinic in Adeoyo Maternity Teaching Hospital and Saint Mary’s Catholic General Hospital, Ibadan, Oyo State. The inclusion criteria were: all the women of reproductive age (15–45 years) living with HIV attending PEPFAR/APIN clinic in Adeoyo maternity teaching hospital and Saint Mary’s hospital who were willing to participate. The exclusion criteria were: all the HIV infected women who were not of reproductive age and those who were not willing to participate.

Knowledge of PMTCT services was measured by percentages. Responses were assigned scores Yes=1 and No=0. The total obtainable mark was 37. The level of knowledge assessment was divided into sections: good knowledge (18–37 marks) and poor knowledge (1–17 marks). The perception of women about PMTCT of HIV programs and services was analyzed using percentages. The variables were coded as follows: strongly agree=5, agree=4, undecided=3, disagree=2 and strongly disagree=1. The total obtainable score was 70. The level of perception assessment was divided into sections: positive perception (45–70 marks) and negative perception (1–44 marks). Utilization of PMTCT service which is integrated into the usual monitoring of pregnant women is measured with the option of Yes or No to the question of ever utilized the services. If a pregnant woman tests positive to HIV and continues with follow-up care it means she is utilizing the services but if she does not comply then it is a non-utilization.

Ethical approval was obtained from UI/UCH Institutional Review Board. Ethical principles were observed. Participants were given a brief and clear introduction on the purpose of the study which is to address their perceptions and utilization of PMTCT services and their consent was obtained. They were assured that confidentiality and anonymity was maintained by informing the respondents that their names should not be included on the questionnaires given to them. They were also given information on the average duration of time required to fill in the questionnaire and the fact that they were free to discontinue at any time.

Data were analyzed using both descriptive (mean, percentages and frequencies) and inferential statistics (chi-squared) at 5% level of significance.

## RESULTS

**Figure 1:**
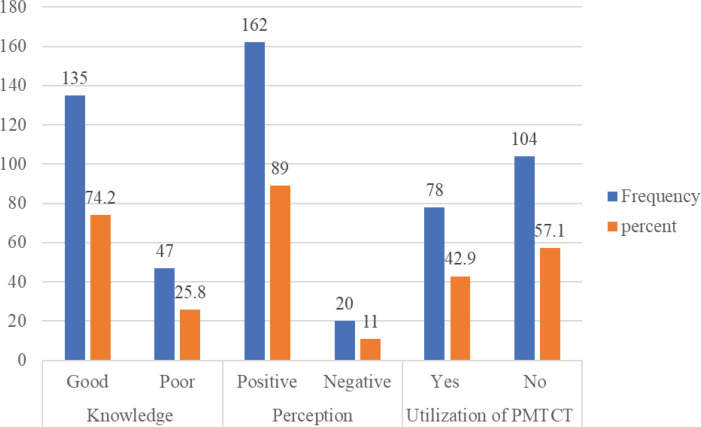
Respondents’ level of knowledge, perception and uptake of PMTCT services

The mean age of the respondents was 37.0±6.5 years, the majority (162; 89%) of the respondents were married, 168 (92.3%) were of Yoruba ethnic group and 151 (83%) were self-employed while many 99 (54.4%) were Muslims (Supplementary Table 1). Figure 1 indicates that the majority (135; 74.2%) had good knowledge on PMTCT of HIV, with a mean score of 24.27±10.1. The majority (162; 89%) of the respondents had positive perception about PMTCT services.

The mean score for perception about PMTCT services was 52.15±9.64. A few respondents identified discrimination (15.4%), financial constraint (11.5%), poor quality of PMTCT services (6.6%) and non-involvement of partner (8.2%) as perceived challenges to non-utilization of PMTCT services. Also, a few of the respondents identified age of mothers (4.9%), occupational status (7.1%), partner’s educational status (3.8%), partner’s HIV status (3.3%), having discussion with partners (2.2%) and client’s satisfaction with the service (6.6%) as factors associated with non-utilization of PMTCT service (Table 1). Only 42.9% of the respondents had utilized PMTCT services during pregnancy. Hypotheses testing showed that that there is a significant association between level of knowledge and utilization of PMTCT services (p=0.012), and knowledge with marital status (Tables 2 and 3)

**Table 1:** Perceived challenges to PMTCT program and services, and factors associated with non-utilization of PMTCT service

	*n (%)*
**Perceived challenges to PMTCT program and services**	
Stigma	30 (16.5)
Discrimination	28 (15.4)
Financial constraints	21 (11.5)
Poor quality of PMTCT services	12 (6.6)
Role of traditional birth attendants	7 (3.8)
Non-involvement of partner	15 (8.2)
Socioeconomic status	7 (3.8)
**Perceived factors with PMTCT service utilization**	
Mother’s age	9 (4.9)
Occupational status	13 (7.1)
Partner’s educational level	7 (3.8)
Partner’s HIV status	6 (3.3)
Having discussions with partner	4 (2.2)
Satisfaction with the service	12 (6.6)

**Table 2:** There is no significant association between selected sociodemographic variables and utilization of PMTCT service

*Variables*	*Utilization of PMTCT services*					
	*Yes*	*No*	*Total*	*χ* ^2^	*df*	*p*
**Age** (years)						
18–24	4	4	8	38.504	3	0.054
25–31	12	15	27			
32–38	35	26	61			
39–45	27	59	86			
**Occupation**						
Unemployed	61	92	153	4.154	2	0.125
Employed	17	12	29			
**Marital status**						
Single	3	8	11	8.623	2	0.013
Married	75	87	162			
Separated	0	9	9			
**Religion**						
Christianity	37	46	83	0.185	1	0.667
Islam	41	58	99			
**Educational level**						
No formal education	4	5	9	0.590	3	0.899
Primary	20	32	52			
Secondary	44	54	98			
Tertiary/university	10	13	23			
**Number of pregnancies**						
<4	67	81	148	6.702	2	0.569
>5	10	13	23			
None	1	10	11			

**Table 3:** Association between utilization of PMTCT services and perceived challenges to PMTCT services

*Perceived challenges*	*Utilization of PMTCT services*				
	*Yes*	*No*	*χ* ^2^	*df*	*p*
**Stigma**			0.421	2	0.810
Yes	23	30			
No	55	72			
**Discrimination**			0.392	2	0.822
Yes	22	28			
No	56	74			
**Financial constraints**			3.943	2	0.139
Yes	26	21			
No	52	81			
**Poor quality of PMTCT services**			0.187	2	0.911
Yes	12	15			
No	66	87			
**Non-involvement of partner**			3.202	2	0.202
Yes	5	72			
No	15	87			
**Socioeconomic factors**			0.636	2	0.728
Yes	8	70			
No	7	92			

## DISCUSSION

PMTCT of HIV services is one of the ways to reduce MTCT of HIV. The findings from this study have shown that respondents demonstrated good knowledge on PMTCT of HIV. This corroborates the findings from similar studies, which revealed that participants in their study had good knowledge of PMTCT services^1,9,17^. However, in contrast to these findings, some studies reported inadequate knowledge of PMTCT of HIV^6,14^. It could be inferred that the good knowledge of PMTCT reported by these women can be associated with the education level of the women as most had secondary and tertiary education. Research has further supported this evidence that educational level and counseling during antenatal periods have aided in improving the knowledge of women on PMTCT services^6,9,18^. Hence, there is a need to improve the knowledge level of women during antenatal clinics through education of PMTCT services and its importance. This will assist in promoting the utilization of the services.

This study revealed that generally, the women had positive perception about PMTCT services. In contrast, a similar study has shown that some respondents have a negative perception towards PMTCT services as they believe the issue is spiritual and needs spiritual intervention^9^. The way an individual perceives his or her health or certain health services or health seeking behaviors will determine to what extent the person is willing to seek or utilize healthcare. This is supported by the Health Belief Model which states that a person’s belief in a personal threat of an illness or disease together with a person’s belief in the effectiveness of the recommended health behavior or action will predict the likelihood that the person will adopt the behaviour^19^. This can be applied in the way PMTCT services are utilized by women living with HIV. The extent women living with HIV perceive PMTCT services as being beneficial or not will determine their willingness to seek and utilize the services. A positive perception towards PMTCT will enhance the utilization of PMTCT services whereas a negative perception will hinder the utilization of PMTCT services. This corroborates the findings of some studies which reported that perception has been associated in the utilization of PMTCT services^9^. It is important that beliefs and several factors that are associated in negative perception of PMTCT services, be dispelled through awareness, education, in order for women to seek PMTCT services.

Furthermore, findings from this study have shown low utilization of PMTCT services during pregnancy. One may think that since the women have good knowledge and positive perception towards PMTCT services, the utilization of PMTCT services amongst these women will be higher, however, only a small percentage utilized these services. A chi-squared test done to ascertain if there is an association between knowledge level and utilization of PMTCT services revealed a significant result. Hence, it is imperative to say that knowledge level is significant in the utilization of PMTCT services. This corroborates the findings of similar studies which showed that knowledge level and educational level had impact on the utilization of PMTCT services^9,15^. Also, perception of PMTCT services has been linked to utilization of the services^9^. Hence, there is a need to dispel negative perceptions amongst participants who have negative perception of PMTCT services in order to enhance the utilization of these services. This can be ensured by counseling and education during antenatal clinics and education through religious leaders on the causes of HIV and its prevention.

Also, certain perceived challenges have been shown in this study to be associated with the utilization of PMTCT services and they are stigma, discrimination, financial constraints, unawareness of HIV seropositive services and poor quality services. This is in agreement with findings of a similar study^11^. The implication of this is that if these challenges are not met, it will lead to low utilization of PMTCT services. Hence, there is a need to find measures to remove these factors, which can be achieved through making the services affordable for the people by government, improving the quality of services, creating awareness to the public on the negative implication of stigmatization and discrimination on the health of these women.

There is also a need to carry out further research to evaluate the effectiveness of PMTCT services in the reduction of mother-to-child-transmission in the facilities, and to compare the perception and utilization PMTCT services of women in urban centers and those resident in rural centers. Exploration of factors influencing utilization PMTCT through a qualitative study will be valuable.

## CONCLUSIONS

Findings suggest there is a need for improvement of knowledge and perception of HIV, MTCT and PMTCT among women through counseling and antenatal education, thereby increasing PMTCT services uptake. Partner involvement, good quality PMTCT services and lack of discrimination of people living with HIV should be encouraged, will help to promote the utilization of PMTCT services.

## Data Availability

The data supporting this research cannot be made available for privacy reasons.
